# Postsynthetic functionalization of glycodendrons at the focal point

**DOI:** 10.3762/bjoc.10.152

**Published:** 2014-07-01

**Authors:** Thisbe K Lindhorst, Katharina Elsner

**Affiliations:** 1 Otto Diels Institute of Organic Chemistry, Christiana Albertina University of Kiel, Otto-Hahn-Platz 3–4, D-24098 Kiel

**Keywords:** amphiphilic glycomimetics, cross metathesis, glycodendrons, multivalent glycoconjugates, multivalent glycosystems

## Abstract

Glycodendrons are multivalent glycoconjugates bearing an orthogonal functional group at the focal point of the molecule. This allows for their postsynthetic elaboration to achieve amphiphilic glycolipid mimetics, for example, which eventually can be applied in biology, biophysics, or material science. Here, postsynthetic modification of di- and tetravalent polyether glycodendrons has been explored using etherification, thiol-ene reaction and in particular olefin cross metathesis.

## Introduction

**Figure 1:**
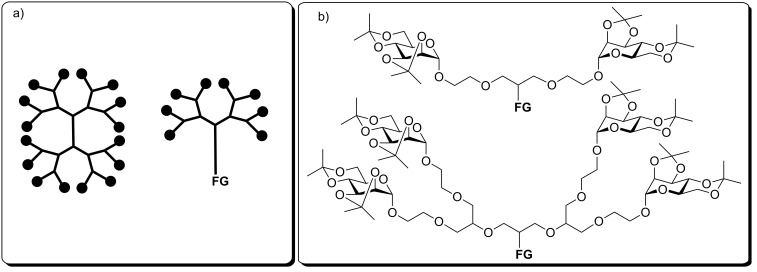
a) Dendrons (right) are branched fragments of dendrimers (left), featuring a functional group (FG) at their focal point which can be orthogonal to all other functionalities of the molecule; b) di- and tetravalent polyether glycodendrons equipped with protected α-D-mannosyl residues were employed to test postsynthetic modification at the focal point; FG: double bond, OH.

In addition to nucleic acids and proteins, molecular life is based on a third important class of compounds, the carbohydrates. Carbohydrates are involved in numerous biological recognition processes, where they are often displayed in the form of multivalent conjugates such as on the surface of cells [1]. To investigate multivalency in carbohydrate recognition, multivalent glycomimetics, for example the glycodendrimers, have become valuable tools during the last two decades [2]. Typical glycodendrimers consist of (hyper)branched dendritic core molecules which are decorated with specific sugars in their periphery [3–5]. In addition to dendrimers, also so-called dendrons have been frequently applied for the synthesis of multivalent glycoconjugates [6]. Dendrons resemble a branched fragment of a whole dendrimer with an orthogonal functional group (FG) at the focal point of the molecular fragment (Figure 1a). This molecular architecture comprises the possibility to anchor a multivalent glycoconjugate to a scaffold or surface, respectively, after suitable postsynthetic modification at the focal point of the molecule. Moreover, such an approach opens the door to a number of intriguing applications of multivalent glycoconjugates such as incorporation into a supramolecular assembly, for example films, liposomes, or membranes.

Focal functionalization of dendrons can be performed prior to modification of the multivalent dendron periphery, or as postsynthetic modification. However, postsynthetic functionalization of the focal point of a rather bulky molecule is not necessarily facile owing to steric hindrance, and therefore has been employed to a lesser extent until to date. Consequently, we have commenced a study on postsynthetic modification of di- and tetravalent polyether glycodendrons, functionalized with a focal double bond or hydroxy group, respectively (Figure 1b).

## Results and Discussion

**Scheme 1:**
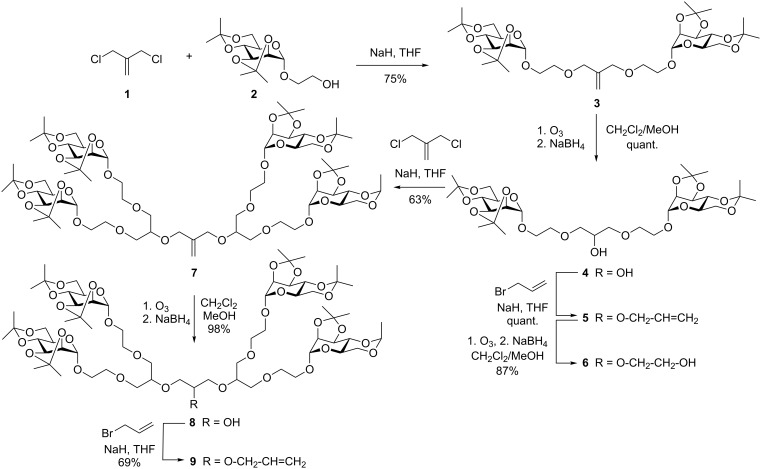
Synthesis of the starting material for postsynthetic focal point functionalization; published yields [7] were partly improved. Glycodendron **9** was obtained for the first time (cf. Experimental part).

The principal synthesis of the employed polyether glycodendrons has been published earlier by us [7–8]. It is based on Williamson etherification of methallyldichloride (MDC, **1**, 3-chloro-2-chloromethyl-1-propene) [9] using the isopropylidene-protected hydroxyethyl mannoside **2** to furnish the divalent glycodendron **3** (Scheme 1). Then, ozonolysis yields the alcohol **4** in a quantitative reaction, which can be further modified at the focal hydroxy group, leading to **5** after allylation and to the primary alcohol **6** in the following ozonolysis step. However, the alcohol **4** can also be employed in another etherification reaction with MDC to deliver glycodendron **7** of the next dendron generation. This in turn, can be further elaborated to give the alcohol **8** and the formerly unknown glycodendron alkene **9**.

**Scheme 2:**
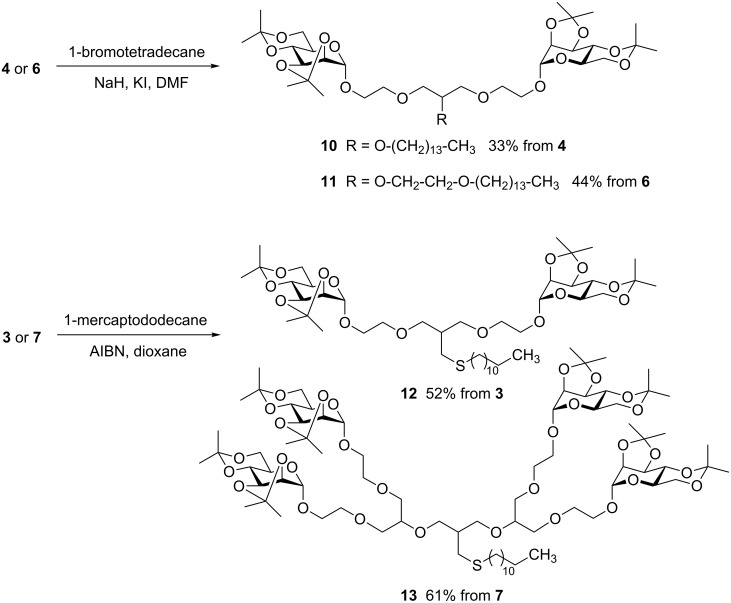
Initial syntheses of amphiphilic glycodendrons.

Initially, postsynthetic focal point modification of glycodendrons was attempted by direct etherification employing long chain alkyl bromides. Williamson etherification of **4** using tetradecanyl bromide led to **10** in only 33% yield, and the same reaction starting with the primary alcohol **6** led to **11** in a somewhat better yield of 44% (Scheme 2). When the tetravalent glycodendron **3** was employed in the same experiment, yields remained below 10%. The focal point apparently is disadvantaged in this reaction. Under those reaction conditions that resulted in at least some yield, degradation of the starting material concomitantly occurred. Also other standard reactions of organic chemistry did not proceed as expected in case of the glycodendrons **3**–**9**. However, the so-called “thiol-ene” reaction [10] gave reliable results with both bivalent and tetravalent glycodendrons. The radical addition of mercaptododecane to either **3** or **7**, employing AIBN as radical starter, led to the amphiphilic thioethers **12** and **13**, respectively, in fair yields. Deprotection conditions employing TFA in water left the thioethers intact. These results were encouraging for further postsynthetic modification of glycodendrons.

**Scheme 3:**
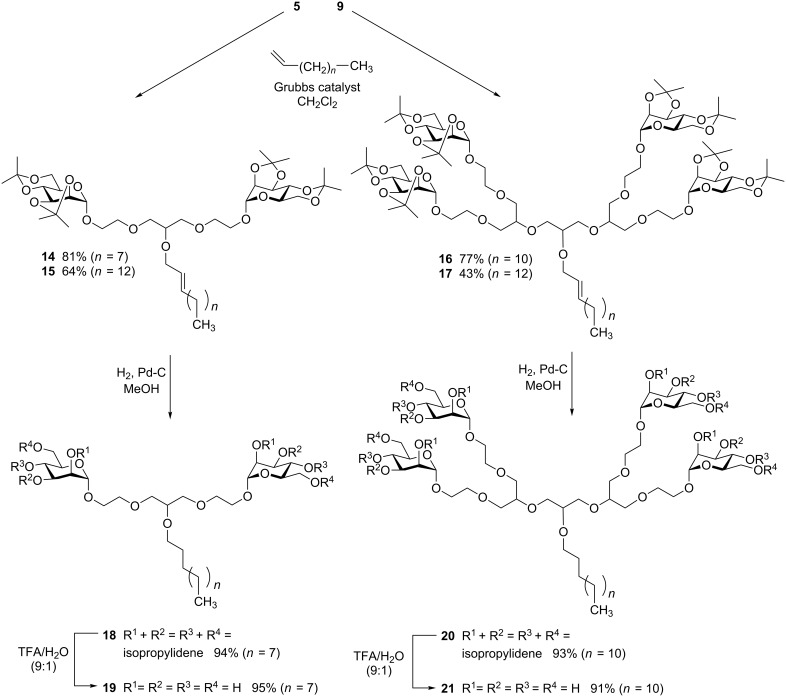
Postsynthetic focal modification of glycodendrons (I) using using olefin cross metathesis.

In a second part of our study we have investigated olefin cross metathesis [11] of polyether di- and tetravalent glycodendrons **5** and **9** with terminal alkenes of different chain length (Scheme 3). Indeed, reaction of **5** and 1-decene using Grubbs’ catalyst (5%) led to the alkene **14** in 81% and the analogous reaction with 1-pentadecene and 10% Grubbs’ catalyst furnished **15** in 64% yield. Interestingly, in both cases, the *trans*-configured alkenes were the only cross-coupling products obtained. This might be due to the specific structure of the used substrates, as sterically hindered olefins are known to enhance *trans*-selectivity in metathesis [12]. The same reactions were successful with the tetravalent glycodendron **9** yielding the cross coupling products **16** and **17** in 77% and 43% respective yields. Again, only the *trans*-metathesis products were obtained. The cross-coupled alkenes **14** and **16** were carried on in catalytic hydrogenation reactions for reduction of the double bond, followed by deprotection of the sugar isopropylidene protecting groups. This supplied the di- and tetravalent amphiphilic glycodendrons **19** and **21**, which can eventually be explored in glycoarray fabrication [13] or in another supramolecular context such as in glycomicelles [14].

**Scheme 4:**
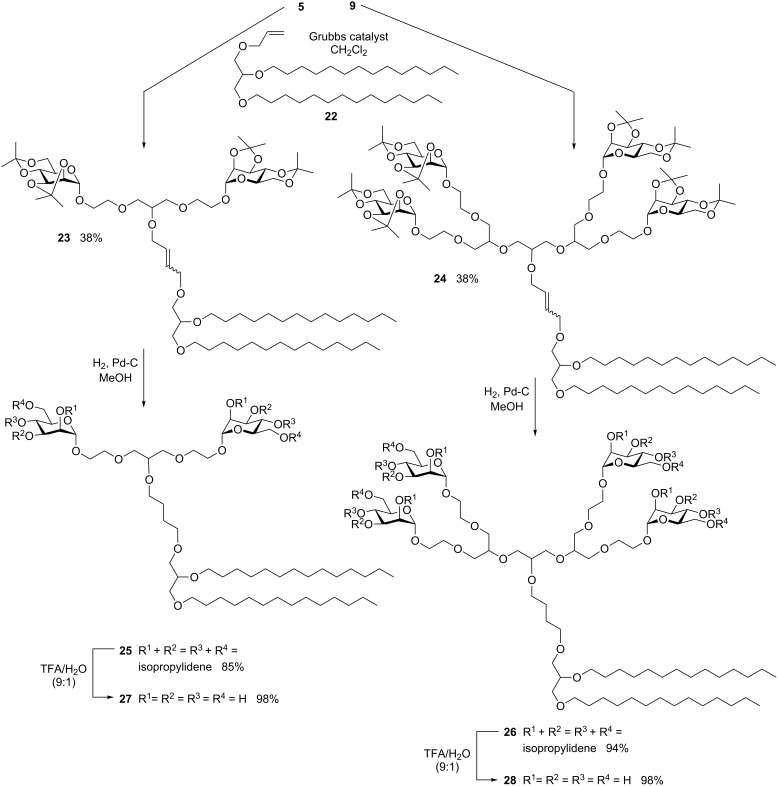
Postsynthetic focal modification of glycodendrons (II) using olefin cross metathesis.

To also achieve the synthesis of branched glycolipid mimetics which are suitable for incorporation into lipid bilayers, the di- and tetravalent glycodendrons **6** and **9** were then cross-coupled with the mono-allylated glycerol triether **22** (Scheme 4). The alkene **22** can be derived from commercially available glycerolmonoallyl ether according to the literature [15]. Metathesis with the divalent glycodendron **6** led to the desired product **23** as *cis*/*trans* mixture in 38% yield, while the glycerol ether dimer was obtained as the main product (not shown). The analogous result was obtained with the tetravalent glycodendron **9** leading to the hetero-cross coupling product **24** as the minor and the homo-cross coupling product as the dominating product. Nevertheless, metathesis allows to achieve these quite complex branched glycolipid mimetics, **23** and **24**, on a multi-100 mg scale. The following hydrogenation of the double bond was carried out in order to resolve the diastereomeric *cis/trans* mixtures leading to the saturated products **25** and **26** in high yields. Then deprotection of the sugar isopropylidene protecting groups furnished the di- and tetravalent amphiphilic glycodendrons **27** and **28**. Purification of the unprotected products was facilitated by gel permeation chromatography (GPC).

## Conclusion

In conclusion, it was shown that readily available polyether glycodendrons can be refined employing suitable postsynthetic modification of the focal point. We have illustrated, that alkylation, thiol-ene reaction and in particular olefin cross metathesis leads to di- and tetravalent glycolipid mimetics that are amenable to a variety of applications, employing Langmuir films [16], self-assembled monolayers (SAMs) [17–19] or lipid bilayers [20], for example. We will eventually optimize some of the described reactions where necessary and validate the described procedures for modification of more complex glycodendrons, including the use of alternative protecting groups. Certainly, thiol-ene and metathesis reaction should be particularly useful also for oligosaccharide glycodendrons, which might be even more sensitive than the herein used molecules.

## Supporting Information

File 1 Detailed experimental procedures and full NMR interpretation of all synthesised compounds.
