# Supplementary material for: Postsynthetic functionalization of glycodendrons at the focal point
Source: Beilstein J Org Chem. 2014 Jul 1;10:1482–7. doi: 10.3762/bjoc.10.152 (PMC4142895; doi:10.3762/bjoc.10.152)
Supplement: File 1 — Detailed experimental procedures and full NMR interpretation of all synthesised compounds. [file Beilstein_J_Org_Chem-10-1482-s001.pdf]

Supporting Information

for

Postsynthetic functionalization of glycodendrons at the focal point

Thisbe K. Lindhorst* and Katharina Elsner

Address: Otto Diels Institute of Organic Chemistry, Christiana Albertina University of Kiel, Otto-Hahn-Platz 3-4, D-24098 Kiel

Email: Thisbe K. Lindhorst* - tklind@oc.uni-kiel.de

*Corresponding author

Detailed experimental procedures and full NMR interpretation of all synthesised compounds.

Experimental

General methods. Reactions requiring dry or oxygen free conditions were carried out under an atmosphere of argon (Schlenk conditions). THF and *n*-hexane were dried by distillation from sodium/potassium ketyl, methanol by distillation from magnesium turnings and dichloromethane by distillation from calcium hydride, each under an atmosphere of argon. Sodium hydride was used as a suspension in paraffin oil (content 55–65%). For metathesis 1st generation Grubbs' catalyst from Aldrich was employed. Thin-layer chromatography was performed on silica gel F_{254} (Merck) or RP-18 (Merck) plates. Detection was effected by UV and subsequent charring with 10% sulfuric acid in EtOH followed by heat treatment. (Flash) chromatography was performed on silica gel 60 (230–400 mesh, particle size 0.040–0.063 mm, Merck) using distilled solvents. Gel permeation chromatography was carried out on Sephadex LH-20 from Pharmacia-Biotech. Sephadex was swollen in the specified solvent and used to pack glass columns of typically 3 cm diameter with bed heights around 100 cm. Sephadex columns were regularly re-used after careful washing. The flow rate was around 50 mL/h using a peristaltic pump. For NMR spectroscopy Bruker DRX 500 or AV 600 instruments were used. Chemical shifts (δ) are calibrated relative to internal solvent. For complete assignment, 2D NMR techniques were used (^{1}H-^{1}H-COSY, ^{1}H-^{13}C-HSQC and ^{1}H-^{13}C HMBC). ESI–MS measurements were performed on a Mariner instrument, MALDI–TOF mass spectra were recorded with a Bruker Biflex III instrument with 19 kV acceleration voltage and an ionization laser at 337 nm; 4-chloro-α-cyanocinnamic acid (Cl-CCA) was used as matrix. Optical rotation values were measured on a Perkin-Elmer 241 polarimeter (10 cm cells, Na-D-line: 589 nm) and are averaged from at least three measurements. Naming of compounds is not according to IUPAC but according to coherent acronyms.

Tetravalent glycodendron(isopropylidene)-*O*-allyl 9

The glycodendron alcohol **8** [1] (226 mg, 163 μmol) was dissolved in dry THF (5 mL) and treated with NaH (60% suspension in paraffin oil, 7 mg, 1.1 equiv) under Schlenk conditions. The reaction mixture was stirred at rt for 5 min, then allyl bromide (18 μL, 212 μmol, 1.3 equiv) was added and the reaction mixture was stirred at 60 °C for 16 h. The mixture was cooled to rt and water (1 mL) and solid potassium carbonate were added for phase separation. The aqueous phase was extracted with ethyl acetate (4 × 10 mL) and the combined organic phases dried over $MgSO_4$. It was filtered and the solvent removed under reduced pressure. Column chromatography (cyclohexane/ethyl acetate, 1:1) delivered the pure product as a colourless oil (161 mg, 113 μmol, 69%); $[\alpha]_D^{20}$ = +5.5 (*c* = 1.1, $CHCl_3$); ^1H NMR (600 MHz, $CDCl_3$): δ = 5.92 (ddt, 1H, O-CH_2-C$\underline{H}$=CH_2), 5.26 (dq, 1H, O-CH_2-CH=C$\underline{H}_2$), 5.15 (dq, 1H, O-CH_2-CH=C$\underline{H}_2$), 5.05 (s, 4H, 4 H-1), 4.20 (d, 4H, $^3J_{2,3}$ = 5.6 Hz, 4 H-2), 4.16 - 4.13 (m, 6H, 44 H-3, OC$\underline{H}_2$CH=CH_2), 3.86 (dd, 4H, $^3J_{5,6}$ = 5.6, $^2J_{6,6'}$ = 10.8 Hz, 4 H-6), 3.80 - 3.76 (m, 6H, C$\underline{H}_2$C$\underline{H}_2$O, 2 $(CH_2)_2$C$\underline{H}$), 3.77 (d, 4H, $^3J_{6,6'}$ = 10.4 Hz, 4 H-6’), 3.74 (dd, 2H, $^3J_{3,4}$ = 7.7, $^3J_{4,5}$ = 10.1 Hz, 4 H-4), 3.67 - 3.53 (m, 29H, 4 H-5, $(CH_2)_2$C$\underline{H}$OCH_2CH=CH_2, C$\underline{H}_2$C$\underline{H}_2$O, 10 CH_2), 1.55, 1.52, 1.42, 1.35 (each s, each 12H, 16 isoprop-CH_3) ppm; ^{13}C NMR (150 MHz, $CDCl_3$): δ = 135.4 ($\underline{C}$H=CH_2), 116.5 ($\underline{C}H_2$=CH), 104.9, 99.7 ($(CH_3)_2\underline{C}$), 97.9 (C-1), 78.6 ($\underline{C}$HOCH_2CH=CH_2), 77.8 ($CH_2\underline{C}$H), 77.2 ($\underline{C}H_2$CH), 76.1 (C-2), 74.9 (C-3), 72.8 (C-4), 71.3 ($\underline{C}H_2$CH=CH_2), 71.1 (2 CH_2), 70.5 (2 CH_2), 70.4 (2 CH_2), 66.7 ($\underline{C}H_2CH_2$O), 62.1 (C-6), 61.4 (C-5), 29.1, 28.2, 26.2, 18.8 (isoprop-CH_3) ppm; HRESI-MS: *m/z*=1448.7229 $[M+Na]^+$ (calcd. *m/z* = 1448.7080) for $C_{68}H_{112}O_{31}$ (M = 1424.7188).

[1] Boysen, M.M.K.; Elsner, K.; Sperling, O.; Lindhorst. T.K. *Eur. J. Org. Chem*. **2003**, 4376-4386.

Divalent glycodendron(isopropylidene)-*O*-tetradecyl 10

The glycodendron **4** (150 mg, 225 μmol) was dissolved in dry DMF (6 mL) and treated with NaH (60% suspension in paraffin oil, 10 mg, 1.1 equiv) under Schlenk conditions. The suspension was stirred at rt for 5 min and then 1-bromotetradecane (68 μL, 250 μmol, 1.1 equiv) and KI (~ 10 mg) were added and the reaction mixture was stirred at 60 °C for 16 h. It was allowed to cool to rt and water (3 mL) was added to quench the reaction. Then, ethyl acetate and water (10 mL each) were added, the phases separated and the aqueous phase extracted with ethyl acetate (4 × 15 mL). The combined organic phases were dried over $MgSO_4$, it was filtered and the filtrate evaporated under reduced pressure. The brownish crude product was purified by column chromatography (cyclohexane/ethyl acetate, 1:1) to yield the title compound as a colourless oil (64 mg, 74 μmol, 33%); $[\alpha]_D^{20}$ = +8.0 (*c* = 0.8, $CHCl_3$); ^1H NMR (500 MHz, $CDCl_3$): δ = 5.06 (s, 2H, 2 H-1), 4.20 (d, 2H, $^3J_{2,3}$ = 5.6 Hz, 2 H-2), 4.14 (dd, 2H, $^3J_{2,3}$ = 5.7, $^3J_{3,4}$ = 7.9 Hz, 2 H-3), 3.87 (dd, 2H, $^3J_{5,6}$ = 5.6, $^2J_{6,6'}$ = 10.8 Hz, 2 H-6), 3.81 - 3.77 (m, 2H, $C\underline{H}_2CH_2O$), 3.75 (d, 2H, $^3J_{6,6'}$ = 10.5 Hz, 2 H-6'), 3.74 (dd, 2H, $^3J_{3,4}$ = 7.9, $^3J_{4,5}$ = 10.1 Hz, 2 H-4), 3.66 - 3.53 (m, 15H, 2 H-5, $(C\underline{H}_2)_2C\underline{H}$, $C\underline{H}_2C\underline{H}_2O$, $CH_2C\underline{H}_2O$, $OC\underline{H}_2(CH_2)_{12}CH_3$), 1.57 ($m_c$, 2H, $OCH_2C\underline{H}_2(CH_2)_{11}CH_3$), 1.55, 1.52, 1.42, 1.35 (each s, each 6H, 8 isoprop-CH_3), 1.32 - 1.24 (m, 22H, $OCH_2CH_2(C\underline{H}_2)_9CH_2$, $C\underline{H}_2CH_2CH_3$, $C\underline{H}_2CH_3$), 0.88 (t, 3H, $^3J_{CH2,CH3}$ = 7.0 Hz, $CH_2C\underline{H}_3$) ppm; ^{13}C NMR (125 MHz, $CDCl_3$): δ = 109.4, 99.7 ($(CH_3)_2\underline{C}$), 97.9 (C-1), 77.8 ($CH_2\underline{C}H$), 76.1 (C-2), 74.9 (C-3), 72.8 (C-4), 71.3 ($O\underline{C}H_2(CH_2)_{11}$), 70.8 (2 CH_2), 70.4 (2 CH_2), 66.7 ($\underline{C}H_2CH_2O$), 62.1 (C-6), 61.3 (C-5), 31.9 ($\underline{C}H_2CH_2CH_3$), 30.1 ($OCH_2\underline{C}H_2(CH_2)_{11}$), 29.7 (3 CH_2), 29.5 (3 CH_2), 29.4 (3 CH_2), 29.1, 28.2, 26.2, 22.7, 18.8 (isoprop-CH_3), ($\underline{C}H_2CH_3$), 14.1 ($CH_2\underline{C}H_3$) ppm; HRESI-MS: *m/z* = 883.5437 $[M+Na]^+$ (calcd. *m/z* = 883.5489) for $C_{45}H_{80}O_{15}$ (M = 860.5497).

Divalent glycodendron(isopropylidene)-*O*-CH_2-CH_2-*O*-tetradecyl 11

The glycodendron **6** (200 mg, 270 μmol) was dissolved in dry DMF (4 mL) under Schlenk conditions and treated with NaH (60% suspension in paraffin oil, 12 mg, 1.1 equiv). It was stirred at RT for 5 min and then 1-bromotetradecane (84 μL, 270 μmol) was added. The reaction mixture was stirred at 60 °C for 16 h, then water (1 mL) was added to quench the reaction and ethyl acetate and water (5 mL each) were added. The phases were separated, and the aqueous phase extracted with ethyl acetate (4 × 10 mL). The combined organic phases were dried over $MgSO_4$, it was filtered and the filtrate evaporated under reduced pressure. Column-chromatographic purification (cyclohexane/ethyl acetate, 3:1) gave the title compound as a colourless oil (107 mg, 118 μmol, 44%); $[\alpha]_D^{20}$ = +2.0 (*c* = 1.0, $CHCl_3$); ^1H NMR (500 MHz, $CDCl_3$): δ = 5.06 (s, 2H, 2 H-1), 4.20(d, 2H, $^3J_{2,3}$ = 5.6 Hz, 2 H-2), 4.15 (dd, 2H, $^3J_{2,3}$ = 5.7, $^3J_{3,4}$ = 8.0 Hz, 2 H-3), 3.86 (dd, 2H, $^3J_{5,6}$ = 5.1, $^2J_{6,6'}$ = 10.6 Hz, 2 H-6), 3.79 (m_c, 2H, $C\underline{H}_2CH_2O$), 3.77 - 3.71 (m, 6H, 2 H-4, 2 H-6', $C\underline{H}_2$), 3.68 (m_c, 1H, $(CH_2)_2C\underline{H}$), 3.66 - 3.59 (m, 14H, 2 H-5, 5 CH_2, $C\underline{H}_2CH_2O$), 3.45 (t, 2H, $OC\underline{H}_2(CH_2)_{12}CH_3$), 1.56 ($m_c$, 2H, $OCH_2C\underline{H}_2(CH_2)_{11}CH_3$), 1.55, 1.52, 1.42, 1.35 (each s, each 6H, 8 isoprop-CH_3), 1.26 (m_c, 22H, $(C\underline{H}_2)_{11}CH_3$), 0.87 (t, 3H, $CH_2C\underline{H}_3$) ppm; ^{13}C NMR (125 MHz, $CDCl_3$): δ = 109.4, 99.7 ($(CH_3)_2\underline{C}$), 97.9 (C-1), 78.4 ($(CH_2)_2\underline{C}H$), 76.0 (C-2), 74.9 (C-3), 72.8 (C-4), 71.6 ($O\underline{C}H_2(CH_2)_{12}CH_3$), 71.5, 71.3, 70.4, 70.3, 70.0 (5 CH_2), 66.6 ($\underline{C}H_2CH_2O$), 62.1 (C-6), 61.3 (C-5), 31.9 ($\underline{C}H_2CH_2CH_3$), 29.7 (2 CH_2), 29.5 (2 CH_2), 29.4 (3 CH_2), 29.1 (2 CH_2, CH_3), 28.2, 26.2, 18.8 (isoprop-CH_3), 22.7 ($\underline{C}H_2CH_3$), 14.1 ($CH_2\underline{C}H_3$) ppm; HRESI-MS: *m/z* = 927.6107 $[M+Na]^+$ (calcd. *m/z* = 927.5652) for $C_{47}H_{84}O_{16}$ (M = 904.5760).

Divalent glycodendron(isopropylidene)-*S*-dodecyl 12

The glycodendron **3** (230 mg, 0.348 mmol) was dissolved in dry dioxane (4 mL) and treated with 1-mercaptododecane (1.26 mL, 5.22 mmol, 15 equiv) and AIBN (25 mg, 0.17 mmol, 0.5 equiv) as radical starter. The reaction mixture was stirred at 60 °C for 4 h. After the reaction was complete (TLC control in cyclohexane/ethyl acetate, 1:1), the solvent was evaporated under reduced pressure. Ethyl acetate and water (10 mL each) were added, the

phases separated and the aqueous phase extracted with ethyl acetate (2 × 5 mL). The combined organic phases were dried over $MgSO_4$, it was filtered and the filtrate evaporated. Chromatographic purification (cyclohexane/ethyl acetate, 3:1), gave the title compound as a colourless oil (157 mg, 0.182 mmol, 52%); $[\alpha]_D^{20}$ = +1.9 (*c* = 0.8, CH_2Cl_2); ^{1}H NMR (500 MHz, $CDCl_3$): δ = 5.06 (s, 2H, 2 H-1), 4.20 (d, 2H, $^3J_{3,4}$ = 5.6 Hz, 2 H-2), 4.15 (dd, 2H, $^3J_{2,3}$ = 5.7, $^3J_{3,4}$ = 7.9 Hz, 2 H-3), 3.86 (dd, 2H, $^3J_{5,6}$ = 5.6, $^2J_{6,6'}$ = 10.7 Hz, 2 H-6), 3.80 - 3.76 (m, 2H, $CH_2C\underline{H}_2O$), 3.75 (t, 2H, $^2J_{6,6'}$ = 10.7 Hz, 2 H-6'), 3.74 (dd, 2H, $^3J_{3,4}$ = 7.9, $^3J_{4,5}$ = 10.1 Hz, 2 H-4), 3.65 - 3.59 (m, 8H, 2 H-5, $C\underline{H}_2CH_2O$, 2 CH_2), 3.57 - 3.50 (m, 4H, 2 CH_2), 2.60 (d, 2H, $^3J_{CH,CH2}$ = 6.7 Hz, $CHC\underline{H}_2S$), 2.50 (t, 2H, $^3J_{CH2,CH2}$ = 7.4 Hz, $SC\underline{H}_2CH_2$), 2.59 (m, 1H, $(CH_2)_2C\underline{H}$), 1.58 (m, 2H, $SCH_2C\underline{H}_2$), 1.55, 1.52, 1.42, 1.35 (each s, each 6H, isoprop-CH_3), 1.29 - 1.25 (m, 18H, $(C\underline{H}_2)_9CH_3$), 0.88 (t, 3H, $^3J_{CH2,CH3}$ = 6.9 Hz, $CH_2C\underline{H}_3$) ppm; ^{13}C NMR (125 MHz, $CDCl_3$): δ = 109.4, 99.7 ($(CH_3)_2\underline{C}$), 97.9 (C-1), 76.1 (C-2), 74.9 (C-3), 72.8 (C-4), 70.7 (OCH_2), 70.1 (OCH_2), 66.6 (OCH_2), 62.1 (C-6), 61.3 (C-5), 39.9 ($\underline{C}HCH_2S$), 33.0 ($S\underline{C}H_2CH_2$), 31.9 ($\underline{C}H_2CH_2CH_3$), 31.3 ($CH\underline{C}H_2S$), 29.7 (4 CH_2), 29.4 (2 CH_2), 29.3 (CH_2), 29.1 (isoprop-CH_3), 28.9 (CH_2), 28.2 (isoprop-CH_3), 26.2 (isoprop-CH_3), 22.7 ($\underline{C}H_2CH_3$), 18.8 (isoprop-CH_3), 14.1 ($CH_2\underline{C}H_3$) ppm; MALDI-ToF-MS: *m/z* = 885.9 $[M+Na]^+$ (calcd. *m/z* = 895.5) for $C_{46}H_{86}O_{14}S$ (M = 862.5).

Tetravalent glycodendron(isopropylidene)-*S*-dodecyl 13

The glycodendron alkene **7** (120 mg, 0.087 mmol) was dissolved in dry dioxane (2 mL) under Schlenk conditions. Then 1-mercaptododecane (0.3 mL 1.3 mmol, 15 equiv) and AIBN (~10 mg) were added and the reaction mixture stirred at 60 °C for 4 h. After the reaction was complete (TLC control in cyclohexane/ethyl acetate, 1:1), the solvent was evaporated under reduced pressure. Ethyl acetate and water (10 mL each) were added, the phases separated and the aqueous phase was extracted with ethyl acetate (2 × 5 mL). The combined organic phases were dried over $MgSO_4$, it was filtered and the filtrate evaporated. Column chromatography on silica gel (cyclohexane/ethyl acetate, 1:1) and subsequent GPC (Sephadex LH-20, eluent MeOH) furnished a colourless oil (84 mg, 0.053 mmol, 61%); $[\alpha]_D^{20}$

= +3.5 (c = 0.8, CH_2Cl_2); ^{1}H NMR (500 MHz, $CDCl_3$): δ = 5.06 (s, 4H, 4 H-1), 4.21 (d, 4H, $^3J_{2,3}$ = 5.6 Hz, 4 H-2), 4.15 (dd, 4H, $^3J_{2,3}$ = 5.7, $^3J_{3,4}$ = 7.7 Hz, 4 H-3), 3.87 (dd, 4H, $^3J_{5,6}$ = 5.6, $^2J_{6,6'}$ = 10.7 Hz, 4 H-6), 3.80 - 3.76 (m, 4H, $C\underline{H}_2C\underline{H}_2O$), 3.75 (d, 4H, $^3J_{6,6'}$ = 10.3 Hz, 4 H-6'), 3.73 (dd, 4H, $^3J_{3,4}$ = 7.5, $^3J_{4,5}$ = 10.1 Hz, 4 H-4), 3.68 - 3.53 (m, 30H, 4 H-5, $CH\underline{H}CH_2O$, $(CH_2)_2C\underline{H}O$, $CH_2C\underline{H}_2O$, $OC\underline{H}_2CHO$, $OC\underline{H}_2CHCH_2$), 2.60 (d, 4H, $^3J_{CH,CH2}$ = 6.6 Hz, $CHC\underline{H}_2S$), 2.48 (t, 2H, $^3J_{CH2,CH2}$ = 7.4 Hz, $SC\underline{H}_2CH_2$), 1.71 (m, 2H, $SCH_2C\underline{H}_2$), 1.55, 1.52, 1.42, 1.35 (each s, each 12H, 16 CH_3), 1.30 - 1.25 (m, 20H, 10 CH_2), 0.88 (t, 3H, $^3J_{CH2,CH3}$ = 7.0 Hz, $CH_2C\underline{H}_3$) ppm; ^{13}C NMR (125 MHz, $CDCl_3$): δ = 109.4, 99.7 ($(CH_3)_2\underline{C}$), 97.8 (C-1), 78.2 ($\underline{C}HCH_2O$), 76.0 (C-2), 74.8 (C-3), 72.7 (C-4), 70.8 (OCH_2), 70.4 (OCH_2), 69.7 (OCH_2), 66.6 (OCH_2), 62.0 (C-6), 61.3 (C-5), 40.3 ($\underline{C}HCH_2S$), 32.8 ($S\underline{C}H_2CH_2$), 31.9 ($\underline{C}H_2CH_2CH_3$), 30.9 ($CH\underline{C}H_2S$), 29.7 (4 CH_2), 29.6, 29.4, 29.3 (3 CH_2), 29.1 (isoprop-CH_3), 29.0 (CH_2), 28.2 (isoprop-CH_3), 26.2 (isoprop-CH_3), 22.7 ($\underline{C}H_2CH_3$), 18.8 (isoprop-CH_3), 14.1 ($CH_2\underline{C}H_3$) ppm; HRESI-MS: m/z = 1605.8606 $[M+Na]^+$ (calcd. m/z = 1605.8573) for $C_{78}H_{134}O_{30}S$ (M = 1582.8681).

Divalent glycodendron(isopropylidene)-*O-trans*-undec-2-enyl 14

The glycodendron **5** (50 mg, 0.071 mmol) was dissolved in dichloromethane (2 mL) under Schlenk conditions and 1-decene (40 µL, 0.28 mmol, 4 equiv) and Grubbs' catalyst (3 mg, 0.004 mmol, 5 mol %) were added. The reaction mixture was stirred under reflux for 16 h. The reaction was quenched by addition of ethylvinyl ether (1 mL), stirred at rt for 45 min and evaporated under reduced pressure. The residual crude product was purified by column chromatography (cyclohexane/ethyl acetate, 1:1) to yield a colourless oil (64 mg, 0.074 mmol, 81%); $[\alpha]_D^{20}$ = +8.0 (c = 0.8, $CHCl_3$); ^{1}H NMR (500 MHz, $CDCl_3$): δ = 5.70 (dt, 1H, $^3J_{CH,CH2}$ = 6.7, $^3J_{CH,CH}$ = 15.3 Hz, $CH{=}C\underline{H}(CH_2)_7CH_3$), 5.56 (dt, 1H, $^3J_{CH,CH2}$ = 6.3, $^3J_{CH,CH}$ = 15.3 Hz, $OCH_2C\underline{H}{=}CH$), 5.06 (s, 2H, 2 H-1), 4.20 (dd, 2H, $^3J_{1,2}$ = 0.8, $^3J_{2,3}$ = 5.6 Hz, 2 H-2), 4.15 (dd, 2H, $^3J_{2,3}$ = 5.7, $^3J_{3,4}$ = 7.9 Hz, 2 H-3), 4.09 (dd, 2H, $^4J_{CH,CH2}$ = 0.9, $^3J_{CH,CH2}$ = 6.3 Hz, $OC\underline{H}_2CH{=}CH$), 3.86 (dd, 2H, $^3J_{5,6}$ = 5.6, $^2J_{6,6'}$ = 10.7 Hz, 2 H-6), 3.79 (m, 2H, 2 $CH\underline{H}CH_2O$), 3.75 (t, 2H, $^3J_{6,6'}$ = 10.5 Hz, 2 H-6'), 3.74 (dd, 2H, $^3J_{3,4}$ = 7.9,

$^3J_{4,5}$ = 10.1 Hz, 2 H-4), 3.68 (m$_c$, 1H, $(CH_2)_2C\underline{H}$), 3.66 - 3.54 (m, 12H, 2 H-5, CH_2CH_2O, $(C\underline{H}_2)_2CH$, $CH_2C\underline{H}_2O$), 2.03 (dq, 2H, $CH{=}CHC\underline{H}_2(CH_2)_6CH_3$), 1.55, 1.52, 1.42, 1.35 (each s, each 6H, 8 isoprop-CH_3), 1.30 - 1.24 (m, 12H, $(C\underline{H}_2)_6CH_3$), 0.98 (t, 3H, $^3J_{CH2,CH3}$ = 7.0 Hz, $CH_2C\underline{H}_3$) ppm; ^{13}C NMR (125 MHz, $CDCl_3$): δ = 134.9 ($CH{=}\underline{C}H(CH_2)_7CH_3$), 126.5 ($OCH_2\underline{C}H{=}CH$), 109.4, 99.7 ($(CH_3)_2\underline{C}$), 97.9 (C-1), 76.6 ($(CH)_2\underline{C}H$), 76.0 (C-2), 74.8 (C-3), 72.8 (C-4), 71.3 ($O\underline{C}H_2CH{=}CH$, CH_2), 70.4 (CH_2), 66.7 ($\underline{C}H_2CH_2O$), 62.1 (C-6), 61.3 (C-5), 32.3 ($CH{=}CHC\underline{H}_2(CH_2)_6CH_3$), 31.9 ($\underline{C}H_2CH_2CH_3$), 29.4 (2 CH_2), 29.3 (2 CH_2), 29.1, 28.2, 26.2 (isoprop-CH_3), 22.7 ($\underline{C}H_2CH_3$),18.8 (isoprop-CH_3), 14.4 ($CH_2\underline{C}H_3$) ppm; HRESI-MS: *m/z* = 841.4988 $[M+Na]^+$ (calcd. *m/z* = 839.4763) for $C_{42}H_{72}O_{15}$ (M = 816.4871).

Divalent glycodendron(isopropylidene)-*O*-*trans*-hexadec-2-enyl 15

The glycodendron **5** (50 mg, 71 µmol) was dissolved in dry dichloromethane (4 mL) under Schlenk conditions and 1-pentadecene (77 µL, 280 µmol, 4 equiv) and Grubbs' catalyst (6 mg, 7 µmol, 10 mol %) were added. The reaction mixture was stirred at 40 °C for 4 h and then quenched by addition of diisopropylamine (1 mL). It was stirred at rt for 40 min, evaporated and the crude product purified by column chromatography (cylohexane/ethyl acetate, 1:1) to yield a colourless oil (40 mg, 45 µmol, 64%); $[\alpha]_D^{20}$ = +3.2 (*c* = 1.0, $CHCl_3$); ^{1}H NMR (600 MHz, $CDCl_3$): δ = 5.69 (ddt, 1H, $^4J_{CH,CH2}$ = 1.0, $^3J_{CH,CH2}$ = 6.7, $^2J_{CH,CH}$ = 15.4 Hz, $CH{=}C\underline{H}CH_2)_{12}CH_3$), 5.55 (dtt, 1H, $^4J_{CH,CH2}$ = 1.4, $^3J_{CH,CH2}$ = 6.3, $^2J_{CH,CH}$ = 15.4 Hz, $OCH_2C\underline{H}{=}CH$), 5.06 (s, 2H, 2 H-1), 4.20 (dd, 2H, $^3J_{1,2}$ = 1.3, $^3J_{2,3}$ = 5.7 Hz, 2 H-2), 4.14 (dd, 2H, $^3J_{2,3}$ = 5.7 Hz, $^3J_{3,4}$ = 7.9 Hz, 2 H-3), 4.09 (dd, 2H, $^4J_{CH,CH2}$ = 1.0, $^3J_{CH,CH2}$ = 6.3 Hz, $OC\underline{H}_2CH{=}CH$), 3.86 (dd, 2H, $^3J_{5,6}$ = 5.7, $^2J_{6,6'}$ = 10.8 Hz, 2 H-6), 3.79 (m, 2H, $C\underline{H}_2CH_2O$), 3.75 (t, 2H, $^2J_{6,6'}$ = 10.7 Hz, 2 H-6'), 3.73 (dd, 2H, $^3J_{3,4}$ = 7.9, $^3J_{4,5}$ = 10.2 Hz, 2 H-4), 3.68 - 3.57 (m, 11H, $(CH_2)_2C\underline{H}$, $C\underline{H}_2CH_2O$, 4 CH_2), 3.55 (dd, 2H, $^3J_{4,5}$ = 5.5, $^3J_{5,6}$ = 10.3 Hz, 2 H-5), 2.03 (m, 2H, $CH{=}CHC\underline{H}_2CH_2$), 1.55, 1.51, 1.42 (each s, each 6H, 6 isoprop-CH_3), 1.38 (m, 2H, $CH{=}CHCH_2C\underline{H}_2(CH_2)_{10}CH_3$), 1.35 (s, 6H, 2 isoprop-CH_3), 1.30 - 1.25 (m, 20H, $(C\underline{H}_2)_{10}CH_3$), 0.88 (t, 3H, $^3J_{CH2,CH3}$ = 7.0 Hz, $CH_2C\underline{H}_3$) ppm; ^{13}C NMR (150 MHz, $CDCl_3$): δ = 134.8 ($CH{=}\underline{C}H(CH_2)_{12}$), 126.6 ($OCH_2\underline{C}H{=}CH$), 109.4, 99.7 ($(CH_3)_2\underline{C}$), 98.0 (C-1), 76.7 ($(CH_2)_2\underline{C}H$),

76.1 (C-2), 74.9 (C-3), 72.8 (C-4), 71.4 ($(\underline{C}H_2)_2CH$), 71.3 ($(\underline{C}H_2)_2CH$), 70.5 ($CH_2\underline{C}H_2O$), 70.4 ($CH_2\underline{C}H_2O$), 66.7 ($\underline{C}H_2CH_2O$), 62.1 (C-6), 61.4 (C-5), 32.3 ($CH{=}CH\underline{C}H_2$), 31.9 ($\underline{C}H_2CH_2CH_3$), 29.7 (4 CH_2), 29.6, 29.5, 29.4, 29.3, 29.1 (5 CH_2), 29.1, 28.2, 26.2 (isoprop-CH_3), 22.7 ($\underline{C}H_2CH_3$), 18.8 (isoprop-CH_3), 14.1 ($CH_2\underline{C}H_3$) ppm; HRESI-MS: *m/z* = 909.5657 $[M+Na]^+$ (calcd. *m/z* = 909.5546) for $C_{47}H_{82}O_{15}$ (M = 886.5654).

Tetravalent glycodendron(isopropylidene)-*O*-*trans*-tetradec-2-enyl 16

The tetravalent glycodendron **9** (140 mg, 98.3 μmol) was dissolved in dichloromethane (2 mL) under Schlenk conditions and 1-tridecene (36 μL, 197 μmol, 2 equiv) and Grubbs' catalyst (8 mg, 10 μmol, 10 mol %) were added. The reaction mixture was stirred under reflux for 16 h. The reaction was quenched by addition of ethyl vinyl ether (1 mL), stirred at rt for 45 min and evaporated under reduced pressure. The residual crude product was purified by column chromatography (cyclohexane/ethyl acetate, 1:1) to yield a colourless oil (118 mg, 75 μmol, 77%); $[\alpha]_D^{20}$ = +5.2 (*c* = 0.6, $CHCl_3$); ^{1}H NMR (500 MHz, $CDCl_3$): = 5.67 (dt, 1H, $^3J_{CH,CH2}$ = 6.7, $^3J_{CH,CH}$ = 15.3 Hz, $CH{=}C\underline{H}CH_2CH_2)_{10}CH_3$), 5.58 (dt, 1H, $^3J_{CH,CH2}$ = 6.2, $^3J_{CH,CH}$ = 15.4 Hz, $OCH_2CH{=}CH$), 5.05 (s, 4H, H-1), 4.20 (d, 4H, $^3J_{2,3}$ = 5.6 Hz, 4 H-2), 4.14 (dd, 4H, $^3J_{2,3}$ = 5.9 Hz, $^3J_{3,4}$ = 7.9 Hz, 4 H-3), 4.07 (d, 2H, $^4J_{CH,CH2}$ = 0.7, $^3J_{CH,CH2}$ = 6.2 Hz, $OC\underline{H}_2CH{=}CH_2$), 3.86 (dd, 4H, $^3J_{5,6}$ = 5.6, $^2J_{6,6'}$ = 10.8 Hz, 4 H-6), 3.80 - 3.76 (m, 4H, CH_2CH_2O), 3.75 (t, 4H, $^2J_{6,6'}$ = 10.5 Hz, 4 H-6'), 3.73 (dd, 4H, $^3J_{3,4}$ = 7.9, $^3J_{4,5}$ = 10.1 Hz, 4 H-4), 3.71 - 3.68 (m, 2H, $(C\underline{H}_2)_2CHOCH_2CH{=}CH$), 3.66 - 3.53 (m, 29H, H-5, $(CH_2)_2C\underline{H}$, $(C\underline{H}_2)_2CHOCH_2CH{=}CH$, $(C\underline{H}_2)_2CH$, $CH\underline{H}CH_2O$, $CH_2C\underline{H}_2O$), 2.02 (m, 2H, $CH{=}CHC\underline{H}_2CH_2$), 1.55, 1.51, 1.42, 1.35 (each s, each 12H, 16 isoprop-CH_3), 1.30 - 1.24 (m, 18H, $(C\underline{H}_2)_9CH_3$), 0.88 (t, 3H, $^3J_{CH2,CH3}$ = 7.0 Hz, $CH_2C\underline{H}_3$) ppm; ^{13}C NMR (150 MHz, $CDCl_3$): δ = 134.4 ($OCH_2\underline{C}H{=}CH$), 126.7 ($CH{=}\underline{C}HCH_2CH_2$), 109.4, 99.7 ($(CH_3)_2\underline{C}$), 97.9 (C-1), 78.6 ($(CH_2)_2\underline{C}H$), 77.4 ($(CH_2)_2\underline{C}H$), 77.2 ($(CH_2)_2\underline{C}H$), 76.1 (C-2), 74.9 (C-3), 72.8 (C-4), 71.1 ($O\underline{C}H_2CH{=}CH$), 70.5, 70.4 (2 CH_2), 66.7 ($\underline{C}H_2CH_2O$), 62.1 (C-6), 61.4 (C-5), 32.4 ($CH{=}CH\underline{C}H_2CH_2$), 31.9 ($C\underline{H}_2CH_2CH_3$), 29.7, 29.6 29.4, 29.2 (4 CH_2), 29.1, 28.2, 26.2 (isopro-CH_3), 22.7 ($C\underline{H}_2CH_3$), 18.8 (isoprop-CH_3), 14.1 ($CH_2\underline{C}H_3$) ppm; HRESI-MS: *m/z* = 1601.8824 $[M+Na]^+$ (calcd.

m/z = 1601.8801), 812.4476 [M+2Na]$^{2+}$ (calcd. *m/z* = 812.4347) for $C_{79}H_{134}O_{31}$ (M = 1578.8909).

Tetravalent glycodendron(isopropylidene)-*O*-*trans*-hexadec-2-enyl 17

The tetravalent glycodendron **9** (100 mg, 70.2 μmol) was dissolved in dry dichloromethane (2 mL) under Schlenk conditions and treated with 1-pentadecene (76 μL, 280 μmol, 4 equiv) and Grubbs' catalyst (3 mg, 3.5 μmol, 5 mol %). The reaction mixture was stirred under reflux for 5 h. The reaction was quenched by addition of ethyl vinyl ether (1 mL), stirred at rt for 45 min and evaporated under reduced pressure. The residual crude product was purified by column chromatography (cyclohexane/ethyl acetate, 1:1) to yield a colourless oil (49 mg, 31.0 μmol, 43%); $[\alpha]_D^{20}$ = +0.5 (*c* = 1.20, $CHCl_3$); ^{1}H NMR (500 MHz, $CDCl_3$): δ = 5.67 (dtt, 1H, $^4J_{CH,CH2}$ = 1.2, $^3J_{CH,CH2}$ = 6.6, $^3J_{CH,CH}$ = 15.4 Hz, $OCH_2CH=C\underline{H}$), 5.62 (dtt, 1H, $^4J_{CH,CH2}$ = 1.4, $^3J_{CH,CH2}$ = 6.2, $^3J_{CH,CH}$ = 15.4 Hz, $C\underline{H}=CH(CH_2)_{12}CH_3$), 5.05 (s, 4H, 4 H-1), 4.20 (d, $^3J_{2,3}$ = 5.7 Hz, 4 H-2), 4.15 (d, $^3J_{3,4}$ = 7.2 Hz, 4 H-3), 4.06 (dd, 1H, $^5J_{CH2,CH2}$ = 1.0, $^3J_{CH,CH2}$ = 6.2 Hz, $OC\underline{H}_2CH=CH$), 3.88 (dd, $^3J_{5,6}$ = 5.6, $^2J_{6,6'}$ = 10.8 Hz, 4H, 4 H-6), 3.79 - 3.70 (m, 10H, 4 H-4, $C\underline{H}_2CH_2O$, 2 CH_2), 3.73 (dd, $^3J_{5,6}$ = 5.7, $^2J_{6,6'}$ = 10.5 Hz, 4H, 4 H-6'), 3.65 - 3.53 (m, 29H, 4 H-5, OCH_2CH_2, 3 CH, 9 CH_2), 2.01 (m, 2H, $C\underline{H}_2CH=CH$), 1.54, 1.51, 1.41 (each s, each 12H, 12 isoprop-CH_3), 1.34 (s, 14H, $OCH_2C\underline{H}_2(CH_2)_{11}CH_3$, 4 isoprop-$CH_3$), 1.27 - 1.24 (m, 20H, $(C\underline{H}_2)_{10}CH_3$), 0.87 (t, 3H, $^3J_{CH2,CH3}$ = 7.0 Hz, $CH_2C\underline{H}_3$) ppm; ^{13}C NMR (125 MHz, $CDCl_3$): δ = 134.5 ($CH=\underline{C}H(CH_2)_{12}CH_3$), 126.6 ($(CH_2)_2OCH_2\underline{C}H=CH$), 109.4, 99.7 ($(CH_3)_2\underline{C}$), 97.9 (C-1), 78.6, 77.4, 77.2 (3 CH), 76.0 (C-2), 74.8 (C-3), 72.8 (C-4), 71.1 ($(CH_2)_2CHO\underline{C}H_2CH=CH$), 71.0 (5 CH_2), 70.4 (5 CH_2), 66.6 ($\underline{C}H_2CH_2O$), 62.1 (C-6), 61.4 (C-5), 32.4 ($CH=CH\underline{C}H_2(CH_2)_{11}CH_3$), 31.9 ($\underline{C}H_2CH_2CH_3$), 29.7 (2 CH_2), 29.6 (3 CH_2), 29.4 (2 CH_2), 29.2 (2 CH_2), 29.1, 28.2, 26.2 (isoprop-CH_3), 22.7 ($\underline{C}H_2CH_3$), 18.8 (isoprop-CH_3), 14.1 ($CH_2C\underline{H}_3$) ppm; MALDI-ToF-MS: *m/z* = 1629.8 [M+Na]$^+$ (calcd. *m/z* = 1629.9), 1645.8 [M+K]$^+$ (calcd. *m/z* = 1645.9) for $C_{81}H_{138}O_{31}$ (M = 1606.9).

Divalent glycodendron(isopropylidene)-*O*-undecyl 18

The alkene **14** (56 mg, 69 μmol) was dissolved in dry MeOH (4 mL), Pd/C (10%, 20 mg) was added and the reaction mixture stirred for 4 h under a hydrogen atmosphere. The course of the reaction was monitored by mass spectrometry (MALDI–TOF). When the reaction was completed, the catalyst was filtered over celite, it was washed with MeOH (5 × 2 mL) and the filtrate evaporated in vacuo. Column chromatographic purification (cyclohexane/ethyl acetate, 1:1) gave the title compound as a colourless oil (36 mg, 54 μmol, 94%); $[\alpha]_D^{20}$ = +3.5 (*c* = 1.5, $CHCl_3$); ^{1}H NMR (600 MHz, $CDCl_3$): δ = 5.05 (s, 2H, 2 H-1), 4.20 (dd, 2H, $^3J_{1,2}$ = 1.4, $^3J_{2,3}$ = 5.7 Hz, 2 H-2), 4.14 (dd, 2H, $^3J_{2,3}$ = 5.7 Hz, $^3J_{3,4}$ = 8.0 Hz, 2 H-3), 3.86 (dd, 2H, $^3J_{5,6}$ = 5.7, $^2J_{6,6'}$ = 10.7 Hz, 2 H-6), 3.80 - 3.77 (m, 2H, $OC\underline{H}_2CH_2$), 3.75 (t, 2H, $^2J_{6,6'}$ = 10.6 Hz, 2 H-6'), 3.73 (dd, 2H, $^3J_{3,4}$ = 8.0, $^3J_{4,5}$ = 10.2 Hz, 2 H-4), 3.66 - 3.52 (m, 15H, 2 H-5, $(C\underline{H}_2)_2C\underline{H}$, OCH_2CH_2, $OCH_2C\underline{H}_2O$), 1.58 - 1.55 (m, 2H, $OCH_2C\underline{H}_2CH_2$), 1.55,1.51, 1.42, 1.35 (each s, each 6H, isoprop-CH_3), 1.32 - 1.25 (m, 16H, $(C\underline{H}_2)_8CH_3$), 0.88 (t, 3H, $^3J_{CH2,CH3}$ = 7.0 Hz, $CH_2C\underline{H}_3$) ppm; ^{13}C NMR (150 MHz, $CDCl_3$): δ = 109.4, 99.7 ($(CH_3)_2\underline{C}$), 97.9 (C-1), 77.9 ($(CH_2)_2\underline{C}H$), 76.1 (C-2), 74.9 (C-3), 72.8 (C-4), 71.2, 70.7, 70.4 (3 CH_2), 66.7 ($O\underline{C}H_2CH_2O$), 62.1 (C-6), 61.4 (C-5), 31.9 ($\underline{C}H_2CH_2CH_3$), 30.1 ($OCH_2\underline{C}H_2CH_2$), 29.7, 29.6, 29.5, 29.4, 29.3 (5 CH_2), 29.1, 28.2, 26.2 (isoprop-CH_3), 26.1 (CH_2), 22.7 ($\underline{C}H_2CH_3$), 18.8 (isoprop-CH_3), 14.1 ($CH_2\underline{C}H_3$) ppm; HRESI-MS: *m/z* = 681.3692 $[M+Na]^+$ (calcd. *m/z* = 681.3668) for $C_{30}H_{58}O_{15}$ (M = 658.3776).

Divalent glycodendron-*O*-undecyl 19

The protected glycodendron **18** (30 mg, 37 μmol) was dissolved in 9:1 TFA–water (5 mL) and the mixture was stirred for ~15 min at rt. After the deprotection reaction was completed (TLC on RP-18 silica gel, acetonitrile/water, 1:1), it was co-distilled with toluene and the residue purified by GPC (Sephadex LH-20, eluent water) to yield a colourless oil (23 mg, 35 μmol, 95%); $[\alpha]_D^{20}$ = +51.3 (*c* = 1.3, CH_3OH); ^{1}H NMR (600 MHz, CD_3OD): δ = 4.84 (s, 2H, H-1), 3.89 - 3.85 (m, 6H, 2 H-2, 2 H-6, CH_2CH_2O), 3.76 (dd, 2H, $^3J_{5,6}$ = 5.7, $^3J_{6,6'}$ = 11.5 Hz, 2 H-6'),

3.76 - 3.74 (m, 2H, 2 H-3), 3.71 - 3.69 (m, 4H, $(C\underline{H}_2)_2CH$, $CH_2C\underline{H}_2O$), 3.68 - 3.65 (m, 4H, H-4, $C\underline{H}_2CH_2O$), 3.64 - 3.57 (m, 9H, 2 H-5, $(CH_2)_2C\underline{H}$, $(C\underline{H}_2)_2CH$, $CH_2C\underline{H}_2O$, $OC\underline{H}_2CH_2CH_2$), 1.60 (quin., 2H, $^3J_{CH2,CH2}$ = 1.2 Hz, $OCH_2C\underline{H}_2(CH_2)_{11}CH_3$), 1.43 - 1.29 (m, 16H, $(C\underline{H}_2)_8CH_3$), 0.94 (t, 3H, $^3J_{CH2,CH3}$ = 7.0 Hz, $CH_2C\underline{H}_3$) ppm; ^{13}C NMR (150 MHz, CD_3OD): δ = 101.7 (C-1), 79.3 ($(CH_2)_2\underline{C}H$), 74.6 (C-5), 72.6 (C-3), 72.1 (C-2), 72.0 (CH_2), 71.7 (CH_2), 71.5 (CH_2), 68.6 (C-4), 67.8 ($\underline{C}H_2CH_2O$), 62.9 (C-6), 33.0 ($\underline{C}H_2CH_2CH_3$), 31.1 ($OCH_2\underline{C}H_2(CH_2)_{11}CH_3$), 30.7 (3 CH_2), 30.6, 30.4, 27.2 (3 CH_2), 23.7 ($\underline{C}H_2CH_3$), 14.1 ($CH_2\underline{C}H_3$) ppm; HRESI-MS: *m/z* = 681.3692 $[M+Na]^+$ (calcd. *m/z* = 681.3668) for $C_{30}H_{58}O_{15}$ (M = 658.3776).

Tetravalent glycodendron(isopropylidene)-*O*-tetradecyl 20

The alkene **16** (45 mg, 29 μmol) was dissolved in dry MeOH (4 mL), Pd/C (10%, 25 mg) was added and the reaction mixture stirred for 4 h under a hydrogen atmosphere. The course of the reaction was monitored by mass spectrometry (MALDI–TOF). When the reaction was completed, the catalyst was filtered over celite, it was washed with MeOH (5 × 2 mL) and the filtrate evaporated in vacuo. Column chromatographic purification (cyclohexane/ethyl acetate, 1:1) gave the title compound as a colourless oil (41 mg, 26 μmol, 93%); $[\alpha]_D^{20}$ = +3.7° (*c* = 1.1, $CHCl_3$); ^{1}H NMR (500 MHz, $CDCl_3$): δ = 5.05 (s, 4H, 4 H-1), 4.20 (dd, 4H, $^3J_{1,2}$ = 1.4, $^3J_{2,3}$ = 5.6 Hz, 4 H-2), 4.15 (dd, 4H, $^3J_{2,3}$ = 5.7 Hz, $^3J_{3,4}$ = 7.9 Hz, 4 H-3), 3.86 (dd, 4H, $^3J_{5,6}$ = 5.7, $^2J_{6,6'}$ = 10.8 Hz, 4 H-6), 3.80 - 3.70 (m, 6H, CH_2CH_2O, $CH_2C\underline{H}_2O$, $(C\underline{H}_2)_2CH$), 3.75 (t, 4H, $^2J_{6,6'}$ = 10.5 Hz, 4 H-6'), 3.74 (dd, 4H, $^3J_{3,4}$ = 7.9, $^3J_{4,5}$ = 10.1 Hz, 4 H-4), 3.66 - 3.53 (m, 31H, H-5, $(CH_2)_2C\underline{H}$, $CH\underline{H}CH_2O$, $CH_2C\underline{H}_2O$, $(C\underline{H}_2)_2CH$, $OC\underline{H}_2(CH_2)_{12}CH_3$), 1.55 (s, 12H, isoprop-CH_3), 1.54 - 1.51 (m, 2H, $OCH_2C\underline{H}_2(CH_2)_{11}CH_3$), 1.51, 1.42, 1.35 (each s, each 12H, isoprop-CH_3), 1.32 - 1.22 (m, 22H, $(C\underline{H}_2)_{11}CH_3$), 0.88 (t, 3H, $^3J_{CH2,CH3}$ = 7.0 Hz, $CH_2C\underline{H}_3$) ppm; ^{13}C NMR (150 MHz, $CDCl_3$): δ = 109.4, 99.7 ($(CH_3)_2\underline{C}$), 97.9 (C-1), 78.5 ($(CH_2)_2\underline{C}H$), 78.3 ($(CH_2)_2\underline{C}H$), 76.0 (C-2), 74.8 (C-3), 72.7 (C-4), 71.0 (2 CH_2), 70.6 (2 CH_2), 70.4 (4 CH_2), 70.3 (2 CH_2), 66.6 ($\underline{C}H_2CH_2O$), 62.0 (C-6), 61.3 (C-5), 31.9 ($C\underline{H}_2CH_2CH_3$), 30.2 ($OCH_2\underline{C}H_2(CH_2)_{11}CH_3$), 29.7 (6 CH_2), 29.6 (CH_2), 29.4 (CH_2), 29.1, 28.2, 26.2 (isoprop-CH_3),

26.9 (CH_2), 22.7 ($C\underline{H}_2CH_3$), 18.8 (isoprop-CH_3), 14.1 ($CH_2\underline{C}H_3$) ppm; HRESI-MS: *m/z* = 1603.8917 $[M+Na]^+$ (calcd. *m/z* = 1603.8958) for $C_{79}H_{136}O_{31}$ (M = 1580.9025).

Tetravalent glycodendron-*O*-tetradecyl 21

The protected glycodendron **20** (22 mg, 14 μmol) was dissolved in 9:1 TFA–water (5 mL) and the mixture was stirred for ~15 min at rt. After the deprotection reaction was completed (TLC on RP-18 silica gel, acetonitrile/water, 1:1), it was co-distilled with toluene and the residue purified by GPC (Sephadex LH-20, eluent water) to yield a colourless oil (16 mg, 13 μmol, 91%); $[\alpha]_D^{20}$ = +51 (*c* = 0.9, CH_3OH); ^1H NMR (600 MHz, CD_3OD): δ = 4.91 (s, 4H, 4 H-1), 3.97 (dd, 4H, $^3J_{1,2}$ = 1.7, $^3J_{2,3}$ = 3.4 Hz, 4 H-2), 3.91 - 3.87 (m, 8H, 4 H-6, CH_2CH_2O), 3.83 (dd, 4H, $^3J_{2,3}$ = 3.3, $^3J_{3,4}$ = 9.6 Hz, 4 H-3), 3.83 - 3.79 (m, 4H, 4 H-6), 3.78 - 3.63 (m, 37H, 4 H-4, 4 H-5, $(C\underline{H}_2)_2C\underline{H}$, $CH_2C\underline{H}_2O$, $OC\underline{H}_2(CH_2)_{12}CH_3$), 1.61 ($m_c$, 2H, $OCH_2C\underline{H}_2(CH_2)_{11}CH_3$), 1.38 - 1.31 (m, 22H, $(C\underline{H}_2)_{11}CH_3$), 0.94 (t, 3H, $^3J_{CH2,CH3}$ = 7.0 Hz, $CH_2C\underline{H}_3$) ppm; ^{13}C NMR (150 MHz, CD_3OD): δ = 101.0 (C-1), 79.1 ($(CH_2)_2\underline{C}H$), 73.8 (C-5), 71.8 (C-3), 71.3 (C-2), 71.2 ($(\underline{C}H_2)_2CH$, $CH_2\underline{C}H_2O$, $O\underline{C}H_2(CH_2)_{12}CH_3$), 67.7 (C-4), 67.4 ($\underline{C}H_2CH_2O$), 62.0 (C-6), 32.8 ($\underline{C}H_2CH_2CH_3$), 30.6 ($OCH_2\underline{C}H_2(CH_2)_{11}CH_3$), 30.5 (7 CH_2), 30.3 (CH_2), 30.2 (CH_2), 26.8 (CH_2), 23.5 ($\underline{C}H_2CH_3$), 14.8 ($CH_2\underline{C}H_3$) ppm; HRESI-MS: *m/z* = 1283.6430 $[M+Na]^+$ (calcd. *m/z* = 1283.6454) for $C_{55}H_{104}O_{31}$ (M = 1260.6562).

Divalent glycodendron(isopropylidene)-*O*-but-2-enyl-glycerol diether 23

The divalent glycodendron **5** (200 mg, 284 μmol) was dissolved in dry dichloromethane (2 mL) under Schlenk conditions and treated with glycerol triether **22** (298 mg 568 μmol, 2 equiv) and Grubbs' catalyst (23 mg, 28 μmol, 10 mol %). The reaction mixture was stirred under reflux for 14 h. The reaction was quenched by addition of ethyl vinyl ether (1 mL), stirred at rt for 30 min and evaporated under reduced pressure. The residual crude product was purified by column chromatography (cyclohexane/ethyl acetate, 1:1) to yield a colourless oil (130 mg, 108 μmol, 38%; 73% *trans* isomer, according to ^1H NMR); $[\alpha]_D^{20}$ = +2.2 (*c* = 0.6, $CHCl_3$); as main product, the cross-coupled homo-dimer of **22** was obtained as a colourless

oil (97 mg, 95 μmol, 81% *trans* isomer). ^{1}H NMR (600 MHz, $CDCl_3$): δ = 5.79 (t, 2H, $^{4}J_{CH,CH2}$ = 2.7 Hz, $CH{=}CH_{trans}$), 5.71 (t, 2H, $^{4}J_{CH,CH2}$ = 5.4 Hz, $CH{=}CH_{cis}$), 5.05 (s, 2H, 2 H-1), 4.22 (d, 2H, $^{3}J_{CH,CH2}$ = 5.3 Hz, $C\underline{H}_2CH{=}CH_{cis}$), 4.20 (dd, 2H, $^{4}J_{2,4}$ = 0.9, $^{3}J_{2,3}$ = 5.6 Hz, 2 H-2), 4.16 (m, 2H, $C\underline{H}_2CH{=}CH_{trans}$), 4.14 (dd, 2H, $^{3}J_{2,3}$ = 5.8 Hz, $^{3}J_{3,4}$ = 7.8 Hz, 2 H-3), 4.07 (d, 2H, $^{3}J_{CH,CH2}$ = 5.3 Hz, $C\underline{H}_2CH{=}CH_{cis}$), 4.00 (m, 2H, $C\underline{H}_2CH{=}CH_{trans}$), 3.86 (dd, 2H, $^{3}J_{5,6}$ = 5.6, $^{2}J_{6,6'}$ = 10.8 Hz, 2 H-6), 3.81 - 3.77 (m, 2H, CH_2CH_2O), 3.75 (t, 2H, $^{2}J_{6,6'}$ = 10.4 Hz, 2 H-6'), 3.73 (dd, 2H, $^{3}J_{3,4}$ = 7.8, $^{3}J_{4,5}$ = 10.1 Hz, 2 H-4), 3.69 - 3.63 (m, 7H, $(CH_2)_2C\underline{H}$, 3 CH_2), 3.63 - 3.54 (m, 11H, 2 H-5, $(CH_2)_2C\underline{H}OCHCH{=}C$, $CH\underline{H}CH_2O$, 3 CH_2), 3.50 (dt, 1H, $^{3}J_{CH2,CH2}$ = 5.2, $^{2}J_{CH2,CH2}$ = 10.2 Hz, $C\underline{H}H$), 3.46 (m, 1H, $CH\underline{H}$), 3.43 (t, 2H, $^{3}J_{CH2,CH2}$ = 6.7 Hz, CH_2), 1.58 - 1.53 (m, 4H, 2 $OCH_2C\underline{H}_2(CH_2)_{11}CH_3$), 1.55, 1.51, 1.42, 1.35 (each s, each 6H, isoprop-CH_3), 1.30 - 1.25 (m, 40H, 2 $(C\underline{H}_2)_{10}CH_3$), 0.88 (t, 6H, $^{3}J_{CH2,CH3}$ = 7.0 Hz, 2 $CH_2C\underline{H}_3$) ppm; ^{13}C NMR (150 MHz, $CDCl_3$): δ = 129.5 ($CH_2\underline{C}H{=}CH$), 109.4, 99.7 ($(CH_3)_2\underline{C}$), 98.0 (C-1), 78.0 ($(CH_2)_2\underline{C}HOCHCH{=}$), 77.2 ($(CH_2)_2\underline{C}H$), 76.1 (C-2), 74.9 (C-3), 72.8 (C-4), 71.7 (CH_2), 71.4 ($\underline{C}H_2CH{=}CH_{trans}$), 71.3 (2 CH_2), 71.0 (CH_2), 70.9 (CH_2), 70.7 (CH_2), 70.6 (2 CH_2), 70.5 ($\underline{C}H_2CH{=}CH_{trans}$), 67.2 ($\underline{C}H_2CH{=}CH_{cis}$), 66.7 ($\underline{C}H_2CH_2O$), 66.2 ($\underline{C}H_2CH{=}CH_{cis}$), 62.1 (C-6), 61.4 (C-5), 31.9 ($\underline{C}H_2CH_2CH_3$), 30.2 ($OCH_2\underline{C}H_2(CH_2)_{11}CH_3$), 29.7 (6 CH_2), 29.5 (4 CH_2), 29.4 (4 CH_2), 29.1, 28.2, 26.2 (isoprop-CH_3), 26.1 (4 CH_2), 22.7 ($\underline{C}H_2CH_3$), 18.8 (isoprop-CH_3), 14.1 ($CH_2\underline{C}H_3$) ppm; HRESI-MS: *m/z* = 1223.8348 $[M+Na]^+$ (calcd. *m/z* = 1223.8367) for $C_{66}H_{120}O_{18}$ (M = 1200.8475).

Tetravalent glycodendron(isopropylidene)-*O*-but-2-enyl-glycerol diether 24

The tetravalent glycodendron **9** (100 mg, 70.2 μmol) was dissolved in dry dichloromethane (2 mL) under Schlenk conditions and treated with glycerol triether **22** (74 mg, 140 μmol, 2 equiv) and Grubbs' catalyst (6 mg, 7 μmol, 10 mol %). The reaction mixture was stirred under reflux for 22 h. The reaction was quenched by addition of ethyl vinyl ether (1 mL), stirred at rt for 60 min and evaporated under reduced pressure. The residual crude product was purified by column chromatography (cyclohexane/ethyl acetate, 1:1) to yield a colourless oil (51 mg, 27 μmol, 38%; 81% *trans* isomer, according to ^{1}H NMR); $[\alpha]_D^{20}$ = +2.2 (*c* = 0.6,

$CHCl_3$); ^{1}H NMR (600 MHz, $CDCl_3$): δ = 5.78 (t, 2H, $^4J_{CH,CH2}$ = 2.8 Hz, $CH{=}CH_{trans}$), 5.68 (m_c, 2H, $CH{=}CH_{cis}$), 5.05 (s, 4H, 4 H-1), 4.20 (d, 2H, $C\underline{H}_2CH{=}CH_{cis}$), 4.19 (d, 4H, $^3J_{2,3}$ = 5.5 Hz, 4 H-2), 4.14 (m_c, 2H, $C\underline{H}_2CH{=}CH_{trans}$), 4.14 (dd, 4H, $^3J_{2,3}$ = 5.5, $^3J_{3,4}$ = 7.9 Hz, 4 H-3), 4.05 (d, 2H, $^3J_{CH,CH2}$ = 5.7 Hz, $C\underline{H}_2CH{=}CH_{cis}$), 3.99 ($m_c$, 2H, $C\underline{H}_2CH{=}CH_{trans}$), 3.86 (dd, 4H, $^3J_{5,6}$ = 5.6, $^2J_{6,6'}$ = 10.6 Hz, 4 H-6), 3.79 - 3.76 (m, 4H, $OCH\underline{H}CH_2$), 3.74 (t, 4H, $^2J_{6,6'}$ = 10.3 Hz, 4 H-6'), 3.72 (dd, 4H, $^3J_{3,4}$ = 7.8, $^3J_{4,5}$ = 10.0 Hz, 4 H-4), 3.70 (m_c, 2H, CH_2), 3.65 - 3.53 (m, 32H, H-5, $(CH_2)_2C\underline{H}$, $OCH\underline{H}CH_2$,10 CH_2), 3.52- 3.41 (m, 6H, 3 CH_2), 1.58- 1.54 (m, 4H, 2 $OCH_2C\underline{H}_2CH_2$), 1.55, 1.51, 1.42, 1.35 (each s, each 12H, isoprop-CH_3), 1.32 - 1.22 (m, 44H, 2 $(C\underline{H}_2)_{11}CH_3$), 0.88 (t, 6H, $^3J_{CH2,CH3}$ = 7.0 Hz, $CH_2C\underline{H}_3$) ppm; ^{13}C NMR (150 MHz, $CDCl_3$): δ = 129.7 (CH=CH), 129.1 (CH=CH), 109.4, 99.7 ($(CH_3)_2\underline{C}$), 98.0 (C-1), 78.6 ($(CH_2)_2\underline{C}H$), 78.0 ($(CH_2)_2\underline{C}H$), 77.9 ($(CH_2)_2\underline{C}H$), 77.2 ($(CH_2)_2\underline{C}H$), 76.1 (C-2), 74.9 (C-3), 72.8 (C-4), 71.7 (CH_2), 71.5 ($\underline{C}H_2CH{=}CH_{trans}$), 71.1 ($CH_2$), 70.7 ($CH_2$), 70.6 ($CH_2$), 70.5 ($CH_2$), 70.4 ($CH_2$), 70.3 ($\underline{C}H_2CH{=}CH_{trans}$), 67.2 ($\underline{C}H_2CH{=}CH_{cis}$), 66.7 ($O\underline{C}H_2CH_2$), 66.2 ($\underline{C}H_2CH{=}CH_{cis}$), 62.1 (C-6), 61.4 (C-5), 31.9 ($\underline{C}H_2CH_2CH_3$), 30.2 ($OCH_2\underline{C}H_2CH_2$), 29.7 ($CH_2$), 29.6 ($CH_2$), 29.4 ($CH_2$), 29.1, 28.2, 26.2 (isoprop-CH_3), 26.1 (CH_2), 22.7 ($\underline{C}H_2CH_3$), 18.8 (isoprop-CH_3), 14.1 ($CH_2\underline{C}H_3$) ppm; HRESI-MS: *m/z* = 1944.1771 $[M+Na]^+$ (calcd. *m/z* = 1944.1935) for $C_{100}H_{176}O_{34}$ (M = 1921.2043).

Divalent glycodendron(isopropylidene)-*O*-butyl-glycerol diether 25

The alkene **23** (136 mg, 113 μmol) was dissolved in dry MeOH (4 mL), Pd/C (10%, 13 mg) was added and the reaction mixture stirred for 1.5 h under a hydrogen atmosphere. The course of the reaction was monitored by mass spectrometry (MALDI–TOF). When the reaction was completed, the catalyst was filtered over celite, it was washed with MeOH (4 × 3 mL) and the filtrate was evaporated in vacuo. Column chromatographic purification (cyclohexane/ethyl acetate, 2:1) gave the title compound as a colourless oil (116 mg, 960 μmol, 85%); $[\alpha]_D^{20}$ = +2.1 (*c* = 0.8, $CHCl_3$); ^{1}H NMR (600 MHz, $CDCl_3$): δ = 5.05 (s, 2H, 2 H-1), 4.20 (dd, 2H, $^3J_{1,2}$ = 1.0, $^3J_{2,3}$ = 5.6 Hz, 2 H-2), 4.14 (dd, 2H, $^3J_{2,3}$ = 5.7 Hz, $^3J_{3,4}$ = 7.9 Hz, 2 H-3), 3.86 (dd, 2H, $^3J_{5,6}$ = 5.7, $^2J_{6,6'}$ = 10.89 Hz, 2 H-6), 3.80 - 3.77 (m, 2H,

CH_2CH_2O), 3.75 (t, 2H, $^2J_{6,6'}$ = 10.3 Hz, H-6’), 3.73 (dd, 2H, $^3J_{3,4}$ = 7.8, $^3J_{4,5}$ = 10.1 Hz, 2 H-4), 3.66 - 3.53 (m, 6H, 2 H-5, $(CH_2)_2C\underline{H}$, $CH\underline{H}CH_2O$), 3.51 - 3.40 (m, 20H, $CH_2C\underline{H}_2O$, $OC\underline{H}_2(CH_2)C\underline{H}_2O$, $(C\underline{H}_2)_2CH$, $OC\underline{H}_2(CH_2)_{12}CH_3$), 1.62 (quin. 4H, $^3J_{CH2,CH2}$ = 3.1 Hz, $OCH_2(C\underline{H}_2)CH_2O$), 1.58 - 1.55 (m, 4H, $OCH_2C\underline{H}_2(CH_2)_{11}CH_3$), 1.55, 1.51, 1.42, 1.35 (each s, each 6H, isoprop-CH_3), 1.32 - 1.25 (m, 44H, 2 $(C\underline{H}_2)_{11}CH_3$), 0.88 (t, 6H, $^3J_{CH2,CH3}$ = 7.0 Hz, $CH_2C\underline{H}_3$) ppm; ^{13}C NMR (150 MHz, $CDCl_3$): δ = 109.4, 99.7 ($(CH_3)_2\underline{C}$), 97.9 (C-1), 78.0 ($(CH_2)_2\underline{C}H$), 77.9 ($(CH_2)_2\underline{C}H$), 76.1 (C-2), 74.9 (C-3), 72.8 (C-4), 71.7, 71.3, 71.2 (3 CH_2), 71.0 (2 CH_2), 70.6, 70.5, 70.4 (3 CH_2), 66.7 ($\underline{C}H_2CH_2O$), 62.1 (C-6), 61.4 (C-5), 31.9 ($\underline{C}H_2CH_2CH_3$), 30.2 ($OCH_2\underline{C}H_2(CH_2)_{11}CH_3$), 29.7 (2 CH_2), 29.5 (2 CH_2), 29.4 (2 CH_2), 29.1, 28.2 (isoprop-CH_3), 26.8 (CH_2), 26.4 ($OCH_2(\underline{C}H_2)_2CH_2O$), 26.2 (isoprop-$CH_3$), 26.2 ($CH_2$), 26.1 ($CH_2$), 22.7 ($\underline{C}H_2CH_3$), 18.8 (isoprop-$CH_3$), 14.1 ($CH_2\underline{C}H_3$) ppm; HRESI-MS: *m/z* = 1225.8504 $[M+Na]^+$ (calcd. *m/z* = 1225.8523) for $C_{66}H_{122}O_{18}$ (M = 1202.8631).

Tetravalent glycodendron(isopropylidene)-*O*-butyl-glycerol diether 26

The alkene **24** (87 mg, 45 μmol) was dissolved in dry MeOH (3 mL), Pd/C (10%, 8 mg) was added and the reaction mixture stirred for 1.5 h under a hydrogen atmosphere. The course of the reaction was monitored by mass spectrometry (MALDI–TOF). When the reaction was completed, the catalyst was filtered over celite, it was washed with MeOH (5 × 2 mL) and the filtrate evaporated in vacuo. Column chromatographic purification (cyclohexane/ethyl acetate, 1:1) gave the title compound as a colourless oil (82 mg, 43 μmol, 94%); $[\alpha]_D^{20}$ = +1.7 (*c* = 0.9, $CHCl_3$); ^1H NMR (600 MHz, $CDCl_3$): δ = 5.05 (s, 4H, 4 H-1), 4.19 (dd, 4H, $^3J_{1,2}$ = 1.0, $^3J_{2,3}$ = 5.7 Hz, 4 H-2), 4.15 (m_c, 4H, 4 H-3), 3.86 (dd, 4H, $^3J_{5,6}$ = 5.6, $^2J_{6,6'}$ = 10.8 Hz, 4 H-6), 3.81 - 3.76 (m, 4H, $C\underline{H}_2C\underline{H}_2O$), 3.75 (t, 4H, $^2J_{6,6'}$ = 10.6 Hz, 4 H-6’), 3.73 (dd, 4H, $^3J_{3,4}$ = 7.8, $^3J_{4,5}$ = 9.9 Hz, 4 H-4), 3.68 (dd, 2H, CH_2), 3.65 - 3.53 (m, 32H, 4 H-5, $(C\underline{H}_2)_2C\underline{H}$, $OCH\underline{H}CH_2$,10 CH_2), 3.48 - 3.41 (m, 8H, 4 CH_2), 1.60 (m_c, 4H, $OCH_2(C\underline{H}_2)_2CH_2O$), 1.56 ($m_c$, 4H, 2 $OCH_2C\underline{H}_2(CH_2)_{11}CH_3$), 1.54, 1.51, 1.42, 1.35 (each s, each 12H, isoprop-CH_3), 1.32 - 1.25 (m, 44H, 2 $(C\underline{H}_2)_{11}CH_3$), 0.88 (t, 6H, $^3J_{CH2,CH3}$ = 7.1 Hz, $CH_2C\underline{H}_3$) ppm; ^{13}C NMR (150 MHz, $CDCl_3$): δ = 109.4, 99.7 ($(CH_3)_2\underline{C}$), 97.9 (C-1), 78.6 ($(CH_2)_2\underline{C}H$), 78.4 ($(CH_2)_2\underline{C}H$), 78.0

($(CH_2)_2\underline{C}H$), 76.1 (C-2), 74.9 (C-3), 72.8 (C-4), 71.7 (CH_2), 71.4 (CH_2), 71.1 (2 CH_2), 70.7 (CH_2), 70.5 (CH_2), 70.4 (2 CH_2), 70.2 (CH_2), 66.7 ($\underline{C}H_2CH_2O$), 62.1 (C-6), 61.4 (C-5), 31.9 ($\underline{C}H_2CH_2CH_3$), 30.2 ($OCH_2\underline{C}H_2(CH_2)_{11}CH_3$), 29.7 (4 CH_2), 29.6, 29.4 (2 CH_2), 29.1, 28.2, 26.2 (isoprop-CH_3), 26.5, 26.2, 26.1 (3 CH_2), 22.7 ($\underline{C}H_2CH_3$), 18.8 (isoprop-CH_3), 14.1 ($CH_2\underline{C}H_3$) ppm; HRESI-MS: *m/z* = 1246.2450 $[M+Na]^+$ (calcd. *m/z* = 1946.2092) and 984.6054, $[M+2Na]^{2+}$ (calcd. *m/z* = 984.5992) for $C_{100}H_{178}O_{34}$ (M = 1923.2200).

Divalent glycodendron-*O*-butyl-glycerol diether 27

The protected glycodendron **25** (116 mg, 96 μmol) was dissolved in 9:1 TFA–water (4 mL) and the mixture stirred for ~15 min at rt. After the deprotection reaction was completed (TLC on RP-18 silica gel, acetonitrile/water, 1:1), it was co-distilled with toluene and the residue purified by GPC (Sephadex LH-20, eluent water) to yield a colourless oil (99 mg, 95 μmol, 98%); $[\alpha]_D^{20}$ = +24.7 (*c* = 0.3, CH_3OH); ^1H NMR (600 MHz, CD_3OD): δ = 4.86 (d, 2H, $^3J_{1,2}$ = 1.8 Hz, 2 H-1), 3.89 - 3.86 (m, 2H, OCH_2CH_2), 3.89 (dd, 2H, $^3J_{1,2}$ = 1.8, $^3J_{2,3}$ = 3.4 Hz, 2 H-2), 3.87 (dd, 2H, $^3J_{5,6}$ = 2.2, $^3J_{6,6'}$ = 11.7 Hz, 2 H-6), 3.78 (dd, 2H, $^3J_{2,3}$ = 3.2, $^3J_{3,4}$ = 9.2 Hz, 2 H-3), 3.77 (dd, 2H, $^3J_{5,6}$ = 5.5, $^3J_{6,6'}$ = 11.8 Hz, 2 H-6), 3.72 - 3.47 (m, 28H, 2 H-4, 2 H-5, $(C\underline{H}_2)_2C\underline{H}$, $OCH\underline{H}CH_2$, $CH_2CH_2C\underline{H}_2O$, $OCH_2C\underline{H}_2$, $OCH_2C\underline{H}_2CH_2$), 1.68 ($m_c$, 4H, $OCH_2C\underline{H}_2C\underline{H}_2CH_2O$), 1.60 (quin., 4H, $^3J_{CH2,CH2}$ = 6.8 Hz, $OCH_2C\underline{H}_2CH_2$), 1.37 - 1.31 (m, 44H, $(C\underline{H}_2)_{11}CH_3$), 0.94 (t, 6H, $^3J_{CH2,CH3}$ = 7.0 Hz, $CH_2C\underline{H}_3$) ppm; ^{13}C NMR (150 MHz, CD_3OD): δ = 101.6 (C-1), 79.2 ($(CH_2)_2\underline{C}H$), 79.1 ($(CH_2)_2\underline{C}H$), 74.4 (C-5), 72.6 (CH_2), 72.5 (C-3), 72.4 (CH_2), 72.0 (C-2, CH_2), 71.9 (2 CH_2), 71.7 (CH_2), 71.5 (CH_2), 71.2 (CH_2), 68.5 (C-4), 67.7 ($\underline{C}H_2CH_2O$), 62.8 (C-6), 33.0 ($\underline{C}H_2CH_2CH_3$), 31.0 ($OCH_2\underline{C}H_2(CH_2)_{11}CH_3$), 30.7 (3 CH_2), 30.5, 30.4, 27.7, 27.3, 27.2, 27.1 (6 CH_2), 23.7 ($\underline{C}H_2CH_3$), 14.4 ($CH_2\underline{C}H_3$) ppm; HRESI-MS: *m/z* = 1065.7199 $[M+Na]^+$ (calcd. *m/z* = 1065.7271) for $C_{54}H_{106}O_{18}$ (M = 1042.7379).

Tetravalent glycodendron-*O*-butyl-glycerol diether 28

The protected glycodendron **26** (82 mg, 43 μmol) was dissolved in 9:1 TFA–water (3 mL) and the mixture stirred for ~20 min at rt. After the deprotection reaction was completed (TLC on RP-18 silica gel, acetonitrile/water, 1:1), it was co-distilled with toluene and the residue purified by GPC (Sephadex LH-20, eluent water) to yield a colourless oil (67 mg, 42 μmol, 98%); $[\alpha]_D^{20}$ = +24.7 (*c* = 0.3, CH_3OH); ^1H NMR (600 MHz, CD_3OD): δ = 4.85 (d, 4H, $^3J_{1,2}$ = 1.8 Hz, 4 H-1), 3.89 - 3.85 (m, 4H, OCH<u>H</u>CH_2), 3.88 (dd, 4H, $^3J_{1,2}$ = 1.8, $^3J_{2,3}$ = 3.4 Hz, 4 H-2), 3.87 (dd, 4H, $^3J_{5,6}$ = 2.3, $^3J_{5,6}$ = 11.7 Hz, 4 H-6), 3.78 - 3.74 (m, 8H, 4 H-3, 4 H-6), 3.73 - 3.61 (m, 40H, 4 H-4, 4 H-5, $(CH_2)_2C\underline{H}$, $CH\underline{H}CH_2O$, 12 CH_2), 1.68 (m_c, 4H, $OCH_2(C\underline{H}_2)_2CH_2O$), 1.60 ($m_c$, 4H, $OCH_2C\underline{H}_2(CH_2)_{11}CH_3$), 1.38 - 1.33 (m, 44H, $(C\underline{H}_2)_{11}CH_3$), 0.94 (t, 6H, $^3J_{CH2,CH3}$ = 7.0 Hz, $CH_2C\underline{H}_3$) ppm; ^{13}C NMR (150 MHz, CD_3OD): δ = 101.7 (C-1), 80.0 ($(CH_2)_2\underline{C}H$), 79.4 ($(CH_2)_2\underline{C}H$), 74.6 (C-5), 72.6 (C-3), 72.1 (C-2, 2 CH_2), 71.8 (2 CH_2), 71.5 (CH_2), 71.1 (CH_2), 68.7 (C-4), 67.8 ($O\underline{C}H_2CH_2O$), 63.0 (C-6), 33.1 ($\underline{C}H_2CH_2CH_3$), 31.2 ($OCH_2\underline{C}H_2(CH_2)_{11}CH_3$), 30.8 (4 CH_2), 30.6, 30.5, 27.9, 27.5, 27.3 (5 CH_2), 23.7 ($\underline{C}H_2CH_3$), 14.4 ($CH_2\underline{C}H_3$) ppm; HRESI-MS: *m/z* = 1625.9543 $[M+Na]^+$ (calcd. *m/z* = 1625.9588) and 824.4842, $[M+2Na]^{2+}$ (calcd. *m/z* = 824.4740) for $C_{76}H_{146}O_{34}$ (M = 1602.9696).
